# The role of annexins in central nervous system development and disease

**DOI:** 10.1007/s00109-024-02443-7

**Published:** 2024-04-19

**Authors:** Zachary B. White, Sindhu Nair, Markus Bredel

**Affiliations:** Department of Radiation Oncology, O’Neal Comprehensive Cancer Center, Heersink School of Medicine, The University of Alabama at Birmingham, Birmingham, AL USA

**Keywords:** Annexins, Neurological disorders, CNS tumors, CNS development

## Abstract

Annexins, a group of Ca^2+^-dependent phospholipid-binding proteins, exert diverse roles in neuronal development, normal central nervous system (CNS) functioning, neurological disorders, and CNS tumors. This paper reviews the roles of individual annexins (A1-A13) in these contexts. Annexins possess unique structural and functional features, such as Ca^2+^-dependent binding to phospholipids, participating in membrane organization, and modulating cell signaling. They are implicated in various CNS processes, including endocytosis, exocytosis, and stabilization of plasma membranes. Annexins exhibit dynamic roles in neuronal development, influencing differentiation, proliferation, and synaptic formation in CNS tissues. Notably, annexins such as ANXA1 and ANXA2 play roles in apoptosis and blood-brain barrier (BBB) integrity. Neurological disorders, including Alzheimer’s disease, multiple sclerosis, and depression, involve annexin dysregulation, influencing neuroinflammation, blood-brain barrier integrity, and stress responses. Moreover, annexins contribute to the pathogenesis of CNS tumors, either promoting or suppressing tumor growth, angiogenesis, and invasion. Annexin expression patterns vary across different CNS tumor types, providing potential prognostic markers and therapeutic targets. This review underscores the multifaceted roles of annexins in the CNS, highlighting their importance in normal functioning, disease progression, and potential therapeutic interventions.

## Introduction

**Fig. 1:**
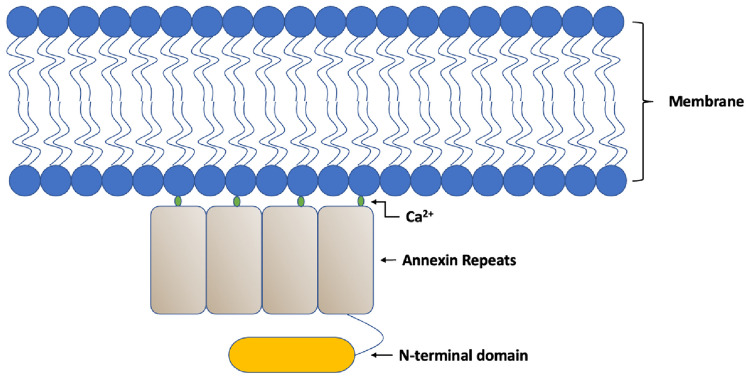
Graphic showing annexin repeats binding in a Ca^2+^-dependent manner to negatively charged phospholipids

Annexins are a group of Ca^2+^-dependent phospholipid-binding proteins. The annexin protein (annex “bring together”) is capable of binding in a Ca^2+^-dependent manner to negatively charged phospholipids. Annexin proteins have been identified in several species, and only 12 out of the over 160 annexin proteins are found in humans. The 12 annexin proteins are referred to as A1-A13, excluding A12, which is currently unassigned. All annexin proteins contain a segment of about 70 amino acid residues called the annexin repeat [1]. Annexins are characterized by their variable N-terminal domain and by their calcium binding site located on the cell membrane [2] (Fig. 1). It contains binding sites for cytoplasmic protein ligands that can be targeted to membranes through the annexin-core-mediated phospholipid interaction [3].

Annexins serve as a scaffold protein when bound to phospholipid membranes. Most annexins are activated and recruited to the cell membrane when calcium is intracellularly released. However, certain annexins (A5, A9, A10) have the ability to bind to membranes in the absence of calcium [1]. The binding of the plasma membrane allows annexins to participate in endocytosis and exocytosis in addition to the stabilization of the plasma membrane. Annexins also can alter the organization of the cell membrane by causing movement of the lipids. This results in the ability to regulate transportation through the membrane via endocytosis and exocytosis [3]. Annexins are traditionally found in the cytosolic side of the plasma membrane; however, some annexins can be found on the extracellular membrane as well. The process of annexins being positioned extracellularly is not fully clear at this point but one study has shown that they function in anti-inflammatory and fibrinolytic activities [4].

Previous research has shown the annexins to be found in genes associated with various central nervous system (CNS) cancers and in neuronal development. Annexin overexpression has been detected in some CNS cancers, such as glioblastoma and astrocytoma. Additionally, the annexin proteins are associated with the development of cortical neurons and the protection of neurons. Many studies have demonstrated that certain annexins could serve as potential therapeutic targets in CNS tumors and neuronal regeneration. In this review, we summarize the role of annexin in neuronal development, neurological disorders within the central nervous system, and CNS tumors (Table 1).

**Table 1:** List of annexins and their cellular roles and functions

**Name**	**Size**	**Role**	**Sources**
**Annexin A1 (ANXA1)**	37 kDa	• cell differentiation, proliferation, plasma membrane repair and apoptosis of a cell	[5]
		• ability to associate with plasma membrane phospholipids, vesicles, and cytoskeleton proteins	
**Annexin A2 (ANXA2)**	36 kDa	• identified in several cell types, including macrophages, endothelial cells, and cancer cells	[6]
		• facilitating the conversion of plasminogen into plasmin	
**Annexin A3 (ANXA3)**	2 isoforms with a molecular mass of 33 and 36 kDa	• endocytosis, transportation of vesicles, and transduction of signals both inside and outside of the cell	[7]
**Annexin A4 (ANXA4)**	35.9 kDa	• promotes vesicle aggregation in vitro, plays a large role in synaptic exocytosis, and has the ability to repair the cell membrane	[8–10]
		• role in calcium signaling, anticoagulation, and resistance to apoptosis	
		• involved in cancer cell proliferation, resistance to chemotherapy, tumor migration, and invasion in various cancer types	
**Annexin A5 (ANXA5)**	36 kDa	• functions in cell membrane repair during anti-inflammatory, profibrinolytic, and anti-thrombotic activities	[1]
		• participates in calcium channel activity	
**Annexin A6 (ANXA6)**	68 kDa	• mediation of endosome aggregation and the secretion of epithelia via exocytosis by vesicle fusion	[11]
**Annexin A7 (ANXA7)**	2 isoforms measuring 47 kDa and 51 kDa	• function in membrane binding and vesicle trafficking	[12, 13]
**Annexin A8 (ANXA8)**	37 kDa	• functions in anticoagulation and phospholipase A_2,_ specifically in the human placenta	[14]
**Annexin A9 (ANXA9)**	36 kDa	• organization and regulation of plasma membrane and cytoskeleton linkage	[15]
**Annexin A10 (ANXA10)**	37 kDa	• calcium- and phospholipid-binding protein	[1]
**Annexin A11 (ANXA11)**	56 kDa	• has the longest N terminus (~ 196 amino acids) of the 12 annexin proteins	[1]
**Annexin A13 (ANXA13)**	36 kDa	• associated with the plasma membrane of proliferating endothelial cells and in the intestinal lining via differentiated villus enterocytes	[16]

## Role of annexins in neuronal development and normal CNS function

ANXA1 has a potential role in the central nervous system. However, the role of ANXA1 in brain development remains uncertain. In the embryos of rats and mice, ANXA1 delineates a midline raphe at the ventral surface of the developing CNS from embryonic day 11 to approximately postnatal day 10 [17, 18]. After progression to adulthood, ANXA1 has reduced neuronal expression throughout the CNS and is found more locally in glia and ependymocytes [17].

ANXA1, a protein initially recognized for its anti-inflammatory actions in peripheral tissues [19], also has a crucial role in normal central nervous system (CNS) function. Microglia, the primary immune cells in the CNS, have been increasingly studied due to their involvement in brain development, aging, and neurodegenerative diseases. Microglia are responsible for coordinating inflammatory responses and efficiently removing apoptotic neurons to maintain brain homeostasis. However, the mechanisms behind the removal of apoptotic cells in the CNS have been unclear. One study highlights the significance of microglial-derived ANXA1 in facilitating the selective removal of apoptotic neurons, a process essential for CNS health [20]. Under noninflammatory conditions, ANXA1 acts as a bridge between microglia and apoptotic neurons, enabling effective clearance of the dying cells while sparing healthy ones. This mechanism involves secreted ANXA1 signaling through the Fpr2 receptor, which is present on microglial cells. This discovery suggests that ANXA1 plays a crucial role in the proactive surveillance of the microglia, rapidly identifying and eliminating apoptotic neurons to maintain CNS integrity.

The expression of ANXA2 is increased in the CNS during the course of an embryo’s development, although the expression levels decline gradually during early postnatal developmental stages [18]. ANXA2 expression is increased in astrocytes of rats after a spinal cord injury, suggesting that ANXA2 might participate in regulating biochemical and physiological responses after a spinal cord injury [21].

In one study, ANXA1, ANXA2, ANXA4, and ANXA6 expressions were examined during development in the CNS of mice [18]. ANXA4 serves as the first annexin to be expressed during the development process. ANXA1, ANXA2, and ANXA4 were found in the floor plate region. ANXA4, in particular, is found present in both the floor and roof plates of the developing CNS. ANXA4 was also highly expressed in dorsal root and sensory ganglion cells. Annexins are therefore associated with the creation of midline structures derived from the floor and roof plates. They are also associated with controlling the decussation of sensory fibers and regulating the processing of sensory neurons. This study underscores that these annexins are not just peripheral players; they are actively involved in essential processes within the CNS such as signal transduction, potential roles in axonal decussation, and interactions with cellular elements like the cytoskeleton and phospholipids [18].

There is evidence that shows ANXA6 functioning as a framework inside the plasma membrane of chick trigeminal and epibranchial sensory neurons [22]. This process is essential for the normal functioning of the cell membrane. Consequently, this suggests that the membrane modifications required to create mature sensory neurons are an ANXA6-dependent process. ANXA6 expression was also noted to be elevated in the neurons of the spinal cord in mice and was also expressed in the sensory neurons of the dorsal root ganglia. Overall, ANXA6 can control Ca^2+^-dependent ionic conductance in both the spinal cord and dorsal root ganglion neurons [23]. To test its neuronal function, the investigators developed an antibody that neutralizes ANXA6-phospholipid interactions and applied it to individual neurons in culture from both spinal cord and dorsal root ganglion neurons. The antibody led to increased K^+^ and Ca^2+^ currents in sensory neurons, indicating that endogenous ANXA6 regulates Ca^2+^ conductance and indirectly affects Ca^2+^-dependent ionic conductances in both the spinal cord and dorsal root ganglion neurons.

Two isoforms of ANXA7 were identified in extracts of human temporal brain tissue, with ANXA7 more specifically expressed in astroglial cells [24]. ANXA7 serves a role in Ca^2+^-dependent signaling in astrocytes [25]. There has also been a focus on the distribution of ANXA7 in the developing brain of mice embryos and in the adult mouse brain. It was demonstrated in the developing brain of mice that expression of Annexin A7 is present during its transportation from the cytoplasm to the nucleus [26]. However, the role of ANXA7 in nuclei of embryonal and mature neuronal cells continues to be an opportunity for future research.

There is currently limited knowledge of ANXA8’s role in neuronal development. However, there is evidence that ANXA8 functions as a key regulator of retinal pigment epithelial (RPE) cell phenotype. ANXA8 is essential to induce neuronal transdifferentiation of RPE cells [27]. RPE cells have several functions that involve the protection and support of the retina. Specifically, they perform phagocytosis of photoreceptor outer segments [28], adsorption of free radicals by pigment granules [29], and formation of the outer blood-retina barrier that maintains ocular immune privilege. [30]

**Fig. 2:**
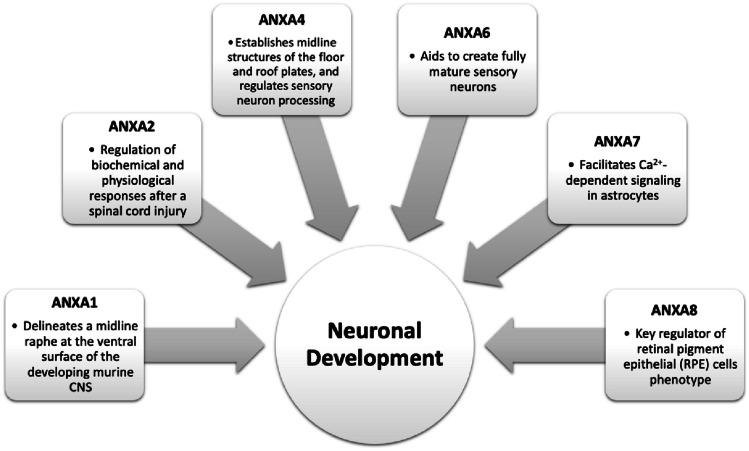
Overview of roles of individual annexins in neuronal development

Overall, several annexins have various roles in neuronal development that involve regulation of biochemical responses after spinal cord injury, establishment of midline structures on the floor and roof plates, and assistance in the development of mature sensory neurons. Their roles have been summarized in Fig. 2. However, there remain annexins that we are still unclear about their role in brain development.

## Role of annexins in neurological disorders of the CNS

Several studies have revealed that annexins play a key role in multiple CNS diseases. Some of the diseases associated with annexins include multiple sclerosis, Alzheimer’s disease, and amyotrophic lateral sclerosis. ANXA1, ANXA2, and ANXA5 have particularly been associated with neurological disorders of the CNS.

Increased expression of ANXA1 was found in various neurological conditions in which the protein was contained entirely in reactive glia or scar tissue. This demonstrates a possible role of ANXA1 in the response to neuronal injury. Elevated ANXA1 levels were identified in the white matter and sclerotic plaques in the brains of patients with multiple sclerosis [31]. It was proposed that increased levels of ANXA1 in patients with multiple sclerosis might demonstrate an ineffective attempt to overcome chronic inflammation. This suggests that a major role of ANXA1 could be involved in both resolving cerebral inflammation and controlling the activity of microglia in brain disease and in healthy states [5]. Additionally, the expression of ANXA1 in brain microvascular endothelial cells plays a vital role in the maintenance of the blood-brain barrier (BBB) integrity. In ANXA1 knockout mice, there was a significant increase in BBB permeability because of disrupted inter-endothelial cell tight junctions [32]. A similar phenomenon was observed in patients with early multiple sclerosis. Human recombinant ANXA1 (hrANXA1) was given intravenously to mice. Consequently, there was a reestablishment of BBB integrity. ANXA1 might therefore be a promising therapeutic addition in the treatment of patients with multiple sclerosis and in correcting BBB function.

ANXA1 could also serve as a potential therapeutic agent in patients diagnosed with Alzheimer’s Disease. Modifications in blood-brain barrier permeability have been noted in patients diagnosed with Alzheimer’s disease [33]. ANXA1 serves as a regulator of blood-brain barrier leakage that can decrease both microvascular damage and amyloid-β pathology [34]. The use of hrANXA1 in mice was also investigated in Alzheimer’s disease. The mice with hrANXA1 had a reversal of cerebrovascular disruption. They also experienced depletion in both amyloid-β pathology and memory loss at an early stage of Alzheimer’s disease, which could yield promising findings in human disease models.

ANXA1 also has neuroendocrine functions within the context of depression. ANXA1, an endogenous ligand of formyl peptide receptor (FPR) 2/3, is recognized for its role in modulating the hypothalamic-pituitary-adrenal (HPA) axis and its association with stress-related psychiatric disorders [35]. The study’s findings indicate that ANXA1 and its receptor play a crucial role in depressive behaviors induced by corticosterone (CORT). Notably, the absence of ANXA1 led to improvements in anxiety and depression-like behaviors, maintenance of hippocampal homeostasis, and prevention of neuronal damage associated with depression. This highlights ANXA1’s intricate involvement in the neuroendocrine regulation of stress responses and its potential as a target for understanding and treating depressive disorders.

One study investigated the neuroprotective potential of chloral hydrate (CH) preconditioning against cerebral ischemic stroke and its reliance on the upregulation of ANXA1 [36]. CH preconditioning is shown to enhance neurological outcomes, reduce infarction and brain edema, and modulate cytokine levels in a middle cerebral artery occlusion (MCAO) model. The role of ANXA1 is demonstrated through experiments utilizing ANXA1 blockers and knockout mice, establishing its necessity for the observed anti-inflammatory and neuroprotective effects. Notably, ANXA1’s influence on microglial function alteration, its anti-inflammatory properties, and its connection to stroke-induced inflammatory pathways are highlighted. Blocking ANXA1 with an antagonist (Boc1) or genetic deletion of ANXA1 eliminated the protective effects of CH against stroke damage, further confirming the role of ANXA1 in CH-mediated neuroprotection against stroke. Overall, CH preconditioning’s neuroprotective actions against cerebral ischemia are attributed to ANXA1 upregulation, suggesting a promising therapeutic avenue for stroke treatment.

ANXA2 has been shown to have a pivotal role in epilepsy. A study demonstrated that ANXA2 plays a significant role in regulating excitatory synaptic activity mediated by the GluA1 subunit of AMPA receptor [37]. Elevated ANXA2 expression was detected in epilepsy patients and models, both in vivo and in vitro. Down-regulating ANXA2 was shown to curb epileptic activity and abnormal neuronal discharges, pointing to its potential antiepileptic effects. The research unveils that ANXA2 primarily influences excitatory synaptic transmission by modulating GluA1 cell-surface protein levels, notably through phosphorylation at specific sites, S831 and S845. The interaction of ANXA2 with crucial signaling pathways involving PKA, PKC, and CaMKII underscores its role in regulating GluA1 phosphorylation. The findings establish ANXA2 as a novel target for potential epilepsy therapies.

ANXA5 is expressed abundantly in small sensory neurons in the dorsal root ganglion. On the contrary, ANXA5 spinal cord expression is high within ependymal cells and in axons of the dorsal horn, specifically in the superficial laminae [38]. Through multiple protein analysis techniques in adult rats, investigators of one study sought to determine the expression of levels of certain annexins following traumatic spinal cord injury. The expression levels of ANXA5 mRNA and protein are increased in the spinal cord after injury [39]. The elevated ANXA5 mRNA expression levels could represent an anti-inflammatory ability to prevent further damage caused by secondary injury. ANXA5 is also important for the development of cortical neurons and the protection of glial cells. It creates a nourishing environment by protecting neurons from hypoxic injuries and peroxide [40].

ANXA10 is upregulated in the spinal cord after spinal nerve ligation and therefore is one of the key molecules in the pathogenesis of neuropathic pain [41]. Ultimately, ANXA10 could be a possible target for therapies of pain management.

**Fig. 3:**
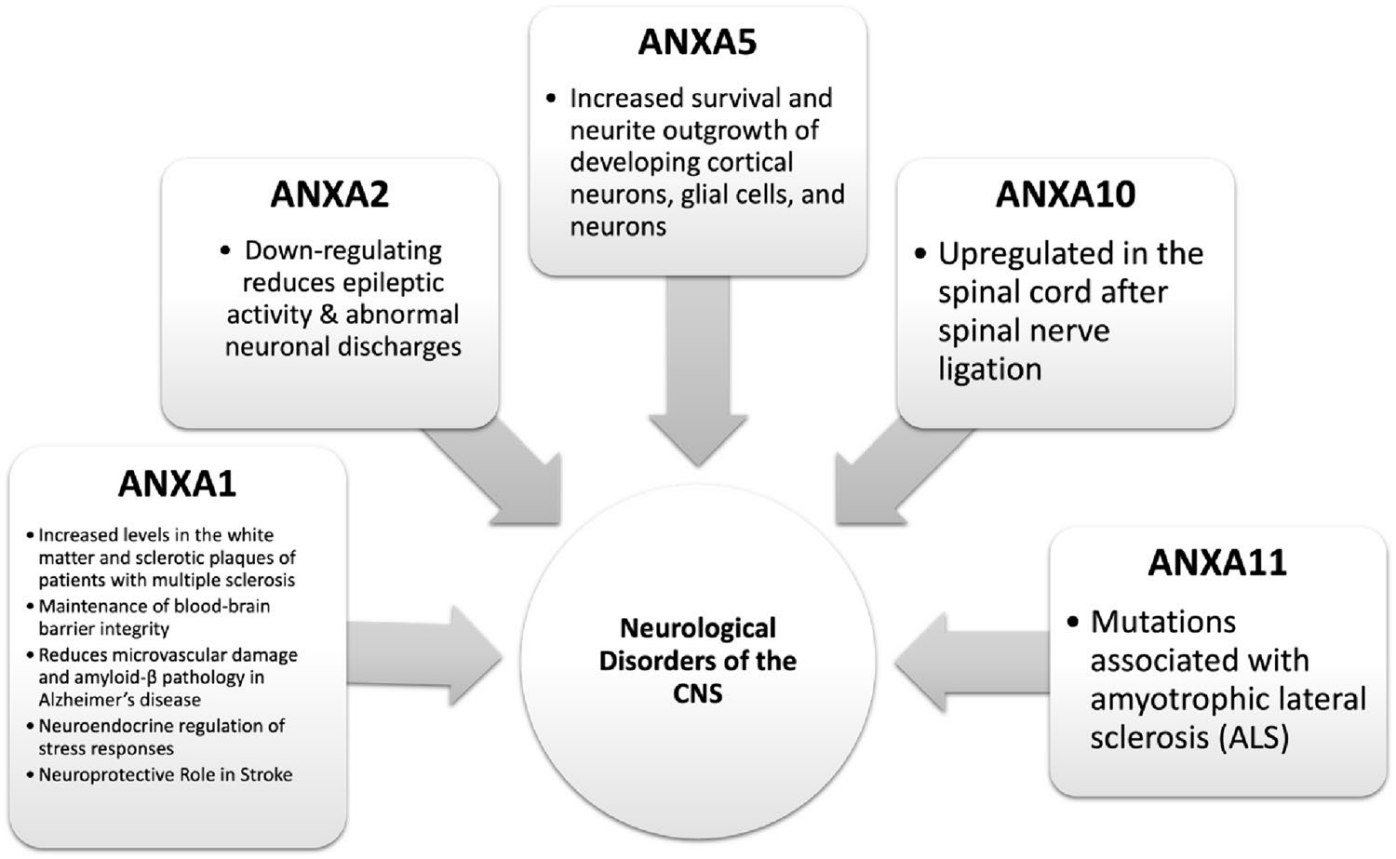
Overview of roles of individual annexins in neurological disorders of the CNS

Mutations in ANXA11 are associated with the fatal neurodegenerative disorder amyotrophic lateral sclerosis (ALS) [42]. The ANXA11 protein is upregulated in patients with ALS and the p.D40G mutation. ANXA11 is particularly found in spinal cord motor neurons and hippocampal neuronal axons. The role of annexins in neurological disease has been summarized in Fig. 3.

There remains future work to be completed to understand more about annexins’ role in CNS diseases such as Alzheimer’s disease, multiple sclerosis, and spinal nerve ligation. Future research could result in therapies for patients who currently have limited treatment options.

## Role of annexins in CNS tumors

Several annexins have been identified to be associated in the development and progression of CNS tumors such as astrocytomas and glioblastomas.

One of the various functions of annexin A1 is the activation of the extracellular signal-regulated kinase (ERK) pathway. ANXA1 can activate this pathway without assistance from nuclear factor-kB. ANXA1 is able to serve as a substrate for the epidermal growth factor receptor (EGFR) and Src-family tyrosine kinases [1, 43, 44].

In human astrocytomas, ANXA1 was shown to be abundantly upregulated in both the cytoplasm and nuclei of the tumor cells. The expression of ANXA1 in normal white and grey matter is restricted to certain brain structures. These include the subependymal astrocytes, a minority of choroid plexus epithelial cells, and the ependymal lining. The expression of ANXA1 was found to be higher in primary glioblastomas as compared to glioblastomas that resulted from the progression of a low-grade astrocytoma, also known as secondary glioblastoma. The ANXA1 substrate EGFR was also found to have high expression in primary glioblastomas [45]. Another study revealed that ANXA1 expression was elevated in glioma tissues compared to normal tissues, and higher ANXA1 expression was associated with poorer prognosis in glioma patients [46]. Investigators utilized various methods, including gene expression analysis from glioma-related databases, Western blot experiments on patient samples, survival analysis, and bioinformatics tools. The findings suggest that ANXA1 overexpression is linked to a worse prognosis in glioma patients and that ANXA1 could serve as a potential therapeutic target for the treatment of gliomas. Additionally, another study found that ANXA1 plays a significant role in the proliferation of glioma cells stimulated by TNF-α [47]. ANXA1 translocated to the cell nucleus in response to TNF-α, influencing the activation of signaling pathways. Importantly, ANXA1 expression correlated with higher-grade gliomas and poor patient prognosis. Collectively, these studies suggest that ANXA1 might have a significant role in the development of high-grade astrocytomas and glioblastomas.

ANXA2 expression has been reported to be increased in diffuse astrocytomas (grades II-IV) when compared with benign pilocytic astrocytoma, which has less tumor infiltration. Additionally, there were reported higher expressions in glioblastoma compared with lower-grade anaplastic astrocytoma or astrocytoma [48]. One study showed that increased levels of ANXA2 in samples of human glioma cells corresponded with increased severity of their disease [49]. ANXA2 was associated with increased features of cancer in glioma cells, such as angiogenesis, migration, and invasion. These features are likely the result of ANXA2 interacting with tissue plasminogen activator (tPA). This results in converting plasminogen to plasmin. Consequently, there is a breakdown of the extracellular matrix and, ultimately, cell invasion. High-grade tumors benefit by having anti-ANXA2 matter localized on the invading margin [49]. A subgroup of glioblastoma patients that have absent or low expression of ANXA2 tend to have a more favorable prognosis with increased progression-free survival and overall survival compared to patients with high expression of ANXA2 [50]. Therefore, inhibition of ANXA2 in gliomas may offer a novel therapeutic strategy and may serve as a possible target in the management of GBM patients. Another study highlighted ANXA2’s role in GBM tumorigenesis [51]. The long non-coding RNA (lncRNA) miR155HG is highly expressed in GBM and acts as a competing endogenous RNA (ceRNA) to “sponge” miR-185-5p, preventing it from inhibiting its target genes. One of the downstream targets of miR-185-5p is ANXA2. ANXA2 is directly regulated by miR-185-5p, and its expression is reduced when miR-185-5p levels are elevated. This establishes the miR155HG/miR-185-5p/ANXA2 axis as a crucial mechanism underlying the malignant behavior of GBM.

ANXA4 expression is increased in various clinical epithelial tumors, including gastric, colorectal, pancreatic, breast, and laryngeal cancers, and gliomas [52]. In particular, ANXA4 is possibly involved in microRNA-7 (miR-7) induced migration and invasion inhibition of glioma [52]. miR-7 is an intronic microRNA that has been shown to promote photoreceptor differentiation and inhibit EGFR and Aky pathway in glioblastoma [53]. The expression of ANXA4 was inhibited 3.5-fold by miR-7 overexpression in glioma cells [54]. This reduction of ANXA4 inhibited migration and invasion capacities of glioma cell lines U251, U87, and A172 and reduced the tumor migration of U251 cells in nude mice. Consequently, it can be inferred that miR-7 suppresses the malignant behavior of glioma cells, at least in part, by impacting the expression of ANXA4.

ANXA5 is associated with the angiogenesis and progression of glioma [55]. Immunodeficient nude rats who underwent xenotransplantation of human gliomas were used for the discovery of biomarkers underlying angiogenic transition. ANXA5 showed a mean increase in non-angiogenic tumors of 2.1-fold and a mean increase in angiogenic tumors of 3.4-fold. ANXA5 was found to have a slightly higher mean increase in angiogenic tumors compared to non-angiogenic tumors [56]. The results suggested that ANXA5 may function in angiogenic tumors through cell-to-cell signaling and enhanced intercellular communication between tumor cells and host cells. This results in the tumor adapting to its microenvironment by causing angiogenesis and cellular proliferation. Overall, ANXA5 could potentially be used as a novel biomarker for high-grade gliomas.

ANXA6 is closely associated with a variety of tumors, including melanoma, breast cancer, gastric cancer, prostate cancer, and epithelial carcinoma; however, no evidence has shown it to be associated with malignancies within the CNS [11].

Available data indicate that ANXA7 might function as a tumor-suppressor gene in glioblastoma. A large majority of glioblastomas demonstrate overactivation of the EGFR pathway as a result of EGFR oncogene mutations [57]. When there is a loss of ANXA7 serving as a tumor suppressor in glioblastoma, this yields to poor control of EGFR signaling and results in a poorer prognosis for the patient [58]. ANXA7 was also found to have an important role in altering the EGFR oncoprotein as a result of lineage-specific splicing [59]. The splicing is managed by the heterogeneous nuclear ribonucleoprotein (hnRNP) polypyrimidine tract–binding protein 1 (PTBP1). PTBP1 had low expression during the normal formation of new neurons to allow maturation of the neuron from progenitor cells [60]. However, PTBP1 is highly expressed in brain tumors [61]. The study aimed to investigate the role of two isoforms of ANXA7 (ANXA7-I1 and ANXA7-I2) in brain tumorigenesis. Expression of ANXA7-I1 mRNA was found to be increased in normal brain tissue compared to in glioblastoma tissue. In contrast, ANXA7-I2 mRNA and protein were found in high amounts in glioblastoma cultures from patients. By employing PTBP1 knockdown in glioblastoma cells, the study observed decreased EGFR protein levels, attenuated EGFR phosphorylation, altered EGFR localization within endosomes, and compromised ERK1/2 activation, and this conclusion was corroborated by concurrent PTBP1 and ANXA7-I1 knockdown, affirming PTBP1’s role in ANXA7-I1 splicing regulation to enhance EGFR signaling and promote tumor proliferation in glioblastoma cells.

Some evidence suggests that ANXA10 could be used as a prognostic biomarker in glioblastoma. ANXA10 was upregulated in glioblastoma, and that correlated with the poor prognosis of the disease [62]. Compared with ANXA1 and ANXA2, which also are overexpressed in glioblastoma, the expression of ANXA10 is lower in glioblastoma. However, ANXA10 had a more remarkable prognostic significance than other ANXAs by performing an univariate analysis.

Understanding more about how the annexins work in the development and progression of CNS tumors could provide the foundation for future selective therapies in these diseases that often present with a poor prognosis.

## Conclusion

**Fig. 4:**
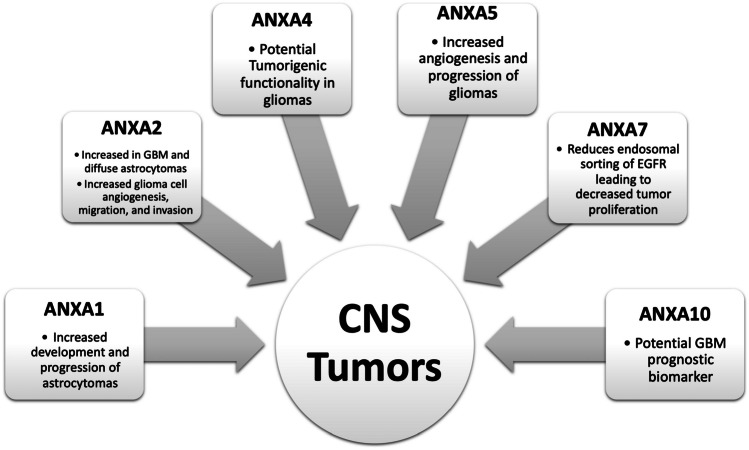
Overview of roles of individual annexins in CNS tumors

Annexins play a wide range of roles, from regulation of biochemical and physiological responses after a spinal cord injury to aiding in the generation of fully mature sensory neurons. However, annexins can have contrasting functions contingent upon pathology. While some annexins are associated with glioma progression, others serve as tumor suppressors. The role of each annexin in neuronal development and disease has been summarized in Figs. 2, 3, and 4. The more we further understand how annexins function in CNS malignancies, CNS disease, and in neuronal development, it can provide the foundation for future selective therapies to reduce tumorigenicity in conditions where currently the outcomes are abysmal.

## Data Availability

Not applicable.
